# Test characteristics and potential impact of the urine LAM lateral flow assay in HIV-infected outpatients under investigation for TB and able to self-expectorate sputum for diagnostic testing

**DOI:** 10.1186/s12879-015-0967-z

**Published:** 2015-07-09

**Authors:** Jonny Peter, Grant Theron, Duncan Chanda, Petra Clowes, Andrea Rachow, Maia Lesosky, Michael Hoelscher, Peter Mwaba, Alex Pym, Keertan Dheda, For the TB-NEAT team

**Affiliations:** Lung Infection and Immunity Unit, Division of Pulmonology & UCT Lung Institute, Department of Medicine, University of Cape Town, Cape Town, South Africa; TB Vaccine group, Jenner Institute, University of Oxford, Oxford, UK; Institute for Medical Research & Training (IMReT), University Teaching Hospital, Lusaka, Zambia; National Institute of Medical Research, Mbeya Medical Research Centre, Mbeya, Tanzania; Division of Infectious Diseases and Tropical Medicine, Medical Centre of the University of Munich (LMU), Munich, Germany; German Centre for Infection Research (DZIF), Munich, Germany; Department of Medicine, University of Cape Town, Cape Town, South Africa; South African Medical Research Council, Durban, South Africa, KwaZulu Research Institute for Tuberculosis and HIV (K-RITH), Durban, South Africa; Institute of Infectious Diseases and Molecular Medicine, University of Cape Town, Cape Town, South Africa

**Keywords:** Tuberculosis, Diagnosis, LAM lateral flow assay, Patient-important impact outcomes

## Abstract

**Background:**

The commercially available urine LAM strip test, a point-of-care tuberculosis (TB) assay, requires evaluation in a primary care setting where it is most needed. There is currently inadequate data to guide implementation in TB and HIV-endemic settings.

**Methods:**

Adult HIV-infected outpatients with suspected pulmonary TB able to self-expectorate sputum from four primary clinics in South Africa, Zambia and Tanzania underwent diagnostic evaluation [sputum smear microscopy, Xpert-MTB/RIF, and culture (reference standard)] as part of a prospective parent study. Urine LAM testing (grade-2 cut-point) was performed on archived samples. Performance characteristics of LAM alone or in combination with sputum—based diagnostics were evaluated. Potential impact on 2 and 6-month morbidity (TBscore), patient dropout rates, and prognosis (death/ loss to follow-up) were evaluated.

**Results:**

Among 583 participants with suspected TB that were HIV-infected or refused testing, the overall LAM sensitivity (95 % CI; n/N) and in the CD4 ≤ 100 cells/mm^3^ sub-group was 22.7 % (16.6-28.7; 41/181) and 30.4 % (17.1-43.7; 14/46), respectively. Overall specificity was 93.0 % (90.5-95.6; 361/388). Amongst culture-positive TB cases, adjunctive LAM testing did not improve the sensitivity of either sputum Xpert-MTB/RIF [78.2 % (69.8-86.7; 72/92) versus 76.1 % (67.4-84.8; 70/92), p = 0.7] or smear-microscopy [56.2 % (45.9-66.5; 50/89) versus 43.8 % (33.5-54.1; 39/89), p = 0.1). Clinic-based LAM, as an adjunct to either smear microscopy or Xpert MTB/RIF same-day testing, would neither have decreased patient dropout, nor increased same-day treatment initiation in this clinical setting where same-day chest radiography was available. LAM positivity was associated with 6-month lost-to-follow-up/death (AOR 4.4; p = 0.002) but not TBscore (at baseline or change in TBscore 2-months post-treatment) (p = 0.17).

**Conclusions:**

In African HIV-TB co-infected outpatients able to self-expectorate sputum LAM had limited sensitivity even at low CD4 counts, and offered no significant incremental diagnostic yield over Xpert-MTB/RIF or smear microscopy. In primary care clinics with chest radiography and where empiric TB treatment is common, LAM seems unlikely to improve rates of same-day treatment initiation and patient dropout, however, the ability of LAM to identify patients at high risk of death or lost-to-follow-up may offer important prognostic value.

**Electronic supplementary material:**

The online version of this article (doi:10.1186/s12879-015-0967-z) contains supplementary material, which is available to authorized users.

## Background

Of the estimated 8.6 million active tuberculosis (TB) cases globally an estimated three million cases remained either undiagnosed or unreported [1]. Thus, tests and technologies that allow rapid, accurate, point-of-care (POC) diagnosis represent an unmet need and are projected to substantially reduce the global TB burden [2, 3]. An increasing number of high TB/HIV burden settings are implementing frontline Xpert MTB/RIF testing for HIV-infected patients with suspected TB [4–6], although sputum smear microscopy remains the frontline TB diagnostic tool in the majority of resource-poor high burden settings. Used at the POC, Xpert MTB/RIF can decrease dropout rates (patients TB-positive but not returning to initiate treatment) and time-to-treatment initiation [5, 7]. However, sensitivity in sputum is reduced in HIV-infected patients [8] and is also suboptimal in induced sputum samples [9]. These and other considerations including infrastructure and electricity requirements means that countries remain interested in simple low-cost non-sputum based POC diagnostic tools for both pulmonary and extrapulmonary TB diagnosis.

In 2013, the Alere Determine™ TB LAM Ag lateral flow strip test (Alere, USA, www.alerehiv.com; referred to as LAM from this point forward) became the first commercially available bedside urine test for TB diagnosis in HIV co-infected patients with results available within 25 minutes using just 60ul of urine [10]. To date, diagnostic accuracy studies of urine LAM have been largely single centre [11–13]. Tested patient populations have been heterogeneous (patients with extrapulmonary TB, those unable to spontaneously provide a sputum sample for TB diagnostic testing, or hospitalised patients) [14] and the incremental value of LAM over sputum smear microscopy or Xpert MTB/RIF has thus been variable [11–17]. Nevertheless, POC tests like urine LAM require evaluation in a primary care settings where they are likely to have most impact and where more than 90 % of the TB case load is first encountered. However, there are limited data to guide implementation in such settings where POC TB tests are most needed. Furthermore, the incremental value of LAM over tests like Xpert MTB/RIF, if any, has hardly been studied in HIV endemic primary care settings. There are also limited published data about the potential impact of LAM on morbidity, same-day treatment initiation, patient dropout rates and prognosis [18, 19].

In this study we sought to provide multicentre comparative accuracy and extrapolate impact data of adjunctive LAM testing in the setting where the overwhelming majority of individuals with presumptive pulmonary TB present (out-patient primary care clinics). These data are needed to make a definitive recommendation about the use of LAM in this key patient population. We hypothesised that in out-patients able to self-expectorate sputum for sputum-based diagnostics, LAM would have very limited incremental utility. We therefore tested the urine for LAM positivity in a cohort of 583 HIV-infected patients with suspected pulmonary TB who formed part of a parent randomised controlled trial comparing Xpert MTB/RIF with same-day smear microscopy in primary care clinics of three sub-Saharan African countries [5]. All the participants provided two expectorated sputa and urine sample.

## Methods

### Design and study population

This cross-sectional accuracy study was nested within a randomised, parallel-arm, multicentre trial, to evaluate the impact of point-of-treatment Xpert MTB/RIF testing with same-day smear microscopy. Patients were enrolled between 12 April 2011 and 1 October 2012. Outpatients ≥18 years that presented to periurban primary-care TB clinics with attached DOTS facilities and microscopy laboratories in Cape Town and Durban (South Africa; laboratory centrally-located), Harare (Zimbabwe), Lusaka (Zambia), and Mbeya (Tanzania) were consecutively enrolled after informed consent. Five Human Research Ethics Committees (HREC) approved the study (University of Cape Town HREC, University of Zambia Biomedical Research Ethics Committee, Mbeya Medical Research Ethics Committee and Medical Research Coordinating Committee of the National Institute for Medical Research, Medical Research Council Durban HREC). Inclusion criteria included: i) symptom(s) of pulmonary TB according to predefined WHO criteria [20, 21] (see online supplementary methods for further details), ii) the ability to spontaneously expectorate two spot sputum specimens with a volume of ≥1 ml each, and exclusion criteria included: i) failure to obtain informed consent and ii) initiation of anti-TB treatment in the previous 60 days. In this sub-study, HIV-uninfected patients were excluded from the analysis. Patients refusing voluntary counselling and testing for HIV (3 %) where considered “positive” and included in the LAM analysis as this would occur in routine clinical practice given the very high (>50 %) incidence of HIV co-infection amongst new TB cases in these endemic countries. A further detailed description of RCT methodology including description of each primary care clinic site is available with the published manuscript of the parent study [5].

### Sample collection and processing

Each patient had at least two spot expectorated sputa collected sequentially at recruitment. Nurses visually inspected expectorated sputum samples and estimated the volume using standards of known volume. Patients randomised to the smear microscopy study arm received two same-day sputum smears for acid-fast bacilli and one arbitrarily selected specimen also underwent culture. Patients in the Xpert MTB/RIF arm received nurse-performed clinic-based Xpert MTB/RIF testing and the other specimen was sent for culture. All patients were asked to provide a spontaneously voided urine sample (10–30ml) into a sterile receptacle. Urine was transferred to the laboratory within 4 hours and frozen at -20 degrees for later batch testing. Urine samples were collected in all parent study sites except Zimbabwe where urine biobanking was not possible.

### Clinical management and follow-up

Patients were enrolled and initially reviewed by research nursing staff. They were offered voluntary testing and counselling for HIV at recruitment, and received a chest radiograph while awaiting their rapid TB test result. TB-related morbidity was assessed at enrolment and during 2- and 6-month follow-up using the previously validated TBscore [22] (Additional file 1 Table S1 in the online supplement). Patients were referred to the local DOTS programme office at the same clinic for the initiation of anti-TB treatment if any positive result from either smear microscopy, Xpert MTB/RIF, or TB culture result was obtained. Smear-or Xpert MTB/RIF negative patients were referred to clinical staff for review together with their chest radiographs and as part of the routine clinic workflow. The WHO guidelines for the treatment of smear-negative TB [21] are routinely used at each clinic. Doctors who were not part of the study team and routinely visited each facility twice a week initiated the treatment of smear- or Xpert MTB/RIF-negative patients. Follow-up was conducted for all study patients by research staff at 2- and 6-months post randomisation (within a range of 14 days before and after both time points). Patients were considered lost-to-follow up if they were not contactable despite multiple attempts at telephonic contact and a community healthcare worker tracing.

### Diagnostic test procedures

Liquid TB culture (Bactec MGIT; BD Microbiology Systems, USA) was performed in central laboratories on sputum decontaminated using *N*-acetyl-L-cysteine–NaOH. Front-loaded, same-day smear microscopy was performed on-site by a technician employed by the programme in a laboratory attached to the healthcare facility, except in South Africa where it was performed at a centralised laboratory. Fluorescence smear microscopy was performed on concentrated samples with auramine-O staining at all sites except Mbeya, Tanzania where concentrated samples underwent ZN-staining and light microscopy. Patients were classified as having smear-positive tuberculosis on the basis of at least one scanty smear (1–9 bacilli per 100 fields [1000× for light microscopy and 400× for fluorescence microscopy]).

Trained staff, including clinical and laboratory staff, according to the manufacturer recommendations on unprocessed thawed urine specimens stored at -20 degrees for not longer than 18 months, performed LAM strip tests. Two independent readers, blinded to reference test results and clinical outcomes, graded (0–5 according to the colour band intensity) each strip after 25–35 minutes using the pre-January 2014 manufacturer’s reference card (see Additional file 1 Figure S1A in supplement). Where results between the two independent readers were discordant, a third consensus reader graded the LAM strip, and this consensus read was used in the analysis. Further details on the reading of LAM strip tests and the reference cards are provided in the online supplement. LAM strip results were neither provided to the clinicians caring for patients nor were they used for treatment decisions.

### Statistical analyses

The reference standard for the primary analysis of diagnostic accuracy was a single sputum liquid culture for *Mycobacterium tuberculosis*. An additional analysis is provided in the online supplement with culture-negative clinical-TB cases considered reference standard positive given the acknowledged limitation of a single sputum TB culture to diagnose TB in HIV co-infection. The study was powered (75–100 % at 95 % confidence interval) to detect differences in diagnostic accuracy between LAM, Xpert MTB/RIF and smear, alone or in combination, for HIV-infected patients. The study was underpowered to detect small differences in diagnostic accuracy measures amongst different CD4 strata. Descriptive statistics were used to characterise the study population. Diagnostic accuracy measures presented include sensitivity, specificity, positive (LR+, PPV) and negative (LR-, NPV) likelihood ratios and predictive values all with 95 % confidence intervals (CI). χ^2^ and Fisher’s exact test with mid-P correction were used for comparisons between proportions, and the Mann-Whitney test was used to compare differences in TBscore. The potential impact of same-day LAM testing was assessed by assuming that LAM testing would be performed at the first point of clinic contact and all LAM-positive patients would initiate anti-TB treatment immediately. χ^2^-squared testing is used to compare same-day treatment initiation and treatment dropout proportions with and without the use of LAM in each study arm. Multivariable- linear (for morbidity scores) and logistic (for mortality) regression analyses were performed. Sample size calculations were based on the primary outcome for the parent study [5] (http://clinicaltrials.gov/show/NCT01554384). Based on the prevalence of HIV and culture-positive TB cases in the parent study there was adequate study power for the 95 % CI around LAM accuracy measures to be within a range of ±10 %. Analyses were performed using OpenEpi (version 2.3.1) [23], and R (version 3.0) [24]. The study is reported in accordance with the STARD initiative recommendations [25].

### Role of the funding source

Alere donated the LAM strip tests. However, neither the company nor the study sponsor had any role in study design, data collection, data analysis, data interpretation, or writing of the report. The corresponding author had full access to all the data in the study and had final responsibility for the decision to submit for publication.

## Results

### Study sites and population characteristics

**Fig. 1:**
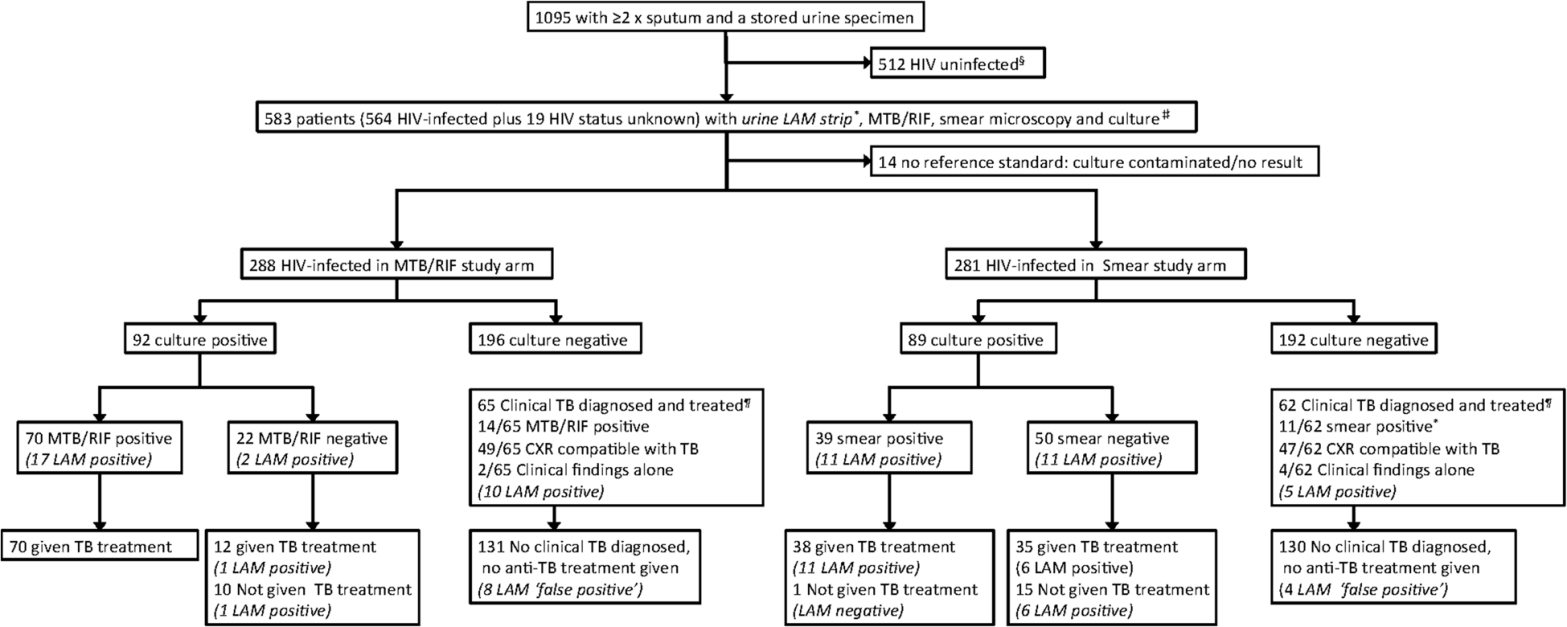
Study profile. ^§^LAM testing is not indicated for HIV uninfected patients. ^#^The 19 patients with refusing HIV testing and hence with unknown HIV status are included with HIV-infected patients for the evaluation of LAM performance and potential impact on TB-related morbidity given that this would occur in routine clinical practice. ^¶^ Clinical TB includes any culture-negative patient given anti-tuberculosis treatment (at any point during the 6 month study period) based on either a sputum-positive Xpert MTB/RIF (n = 14) or smear microscopy (n = 11) result, a Chest x-ray suggestive of TB (n = 96) or clinical suspicion alone (n = 6). ^*^1 Smear-positive culture-negative patient did not receive anti-TB treatment and is thus not considered as “Clinical TB diagnosed and treated”. Results of LAM are provided in italics because they were not used for treatment decisions

Figure 1 provides the study profile. We enrolled 1095 patients with suspected TB, that were able to provide ≥2 sputa and a spot urine sample, from four primary care clinics in South Africa (n = 419 in Cape Town, n = 193 in Durban), Zambia (n = 400) and Tanzania (n = 83). Further detailed descriptions of each primary care clinic have already been published with the parent study [5]. HIV-infection was confirmed in 564/1095 (52 %) and testing was refused in 19/1095 (1 %); LAM test performance is considered in these two groups combined (N = 583). Of the 583 HIV-positive/status unknown patients with suspected TB, 14 (2 %) had either a contaminated culture or no available result, and 181 (31 %) had culture-positive TB. Table 1 provides the basic demographic and clinical characteristics of the HIV-infected patients of the study population stratified by study site.

**Table 1:** Study site and population characteristics

	Gugulethu TB Clinic (Cape Town, South Africa)	Kanyama TB Clinic (Lusaka, Zambia)	St. Mary’s Day Clinic (Durban, South Africa)	Ifisi Day Clinic (Mbeya, Tanzania)	Overall
No. patients enrolled in parent RCT	419	400	193	83	1095
HIV-infected	133 (32)	268 (67)	114 (59)	49 (59)	564 (52)
HIV uninfected	278 (66)	130 (33)	70 (35)	34 (41)	512 (47)
HIV testing refused	8 (2)	2 (<1)	9 (5)	0 (0)	19 (1)
Demographics of HIV-infected/testing refused patients included in primary analysis (*N* = 583)					
	N = 141	N = 270	N = 123	N = 49	N = 583
Median age, years (range, IQR)	35 (19-79, 30-40)	35 (19-79, 30-40)	36 (20-63, 29-42)	36 (17-72, 33-48)	36 (17-79, 30-41)
Women (%)^a^	81 (58)	99 (37)	65 (53)	27 (55)	272 (46)
Previous TB^b^ (%)	69 (49)	42 (16)	37 (30)	2 (4)	150 (26)
Median CD4 cell count, cells/ml (range, IQR)^c^	261 (6-1089, 137-410)	201 (1-1251, 104-339)	231 (2-1157, 106-494)	91 (1-423, 33-213)	210 (1-1251, 103-375)
HIV-infected on ART^d^ (%)	51/133 (38)	54/268 (20)	28/114 (25)	2/49 (4)	135/564 (24)
TB outcomes					
Sputum TB culture-positive patients^e^ (%)	39 (28)	100 (37)	25 (20)	17 (35)	181 (31)
Clinical TB^f^ (%)	17 (12)	26 (10)	6 (5)	4 (8)	53 (9)
TB-related morbidity at baseline in culture-positive patients					
Median TBscore (IQR)^g^	4 (3-5)	6 (4-7)	5 (4-6)	7 (5-9)	5 (4-7)

Abbreviations: *IQR*, interquartile range; *TB*, tuberculosis

Footnotes:^a^A smaller proportion of patients in Lusaka were female compared to Cape Town, Durban and Mbeya (p-values of <0.0001, 0.003, and 0.015)

^b^A greater proportion of patients in Cape Town had a history of previous TB compared to Lusaka, Durban, and Mbeya (*p*-values of <0.0001, 0.002, and <0.0001)

^c^HIV-infected patients in Mbeya had lower CD4 cell counts compared with Cape Town, Lusaka and Durban (p-value of <0.001 for all comparisons)

^d^A greater proportion of HIV-infected patients were on ART in Cape Town compared to Lusaka, Durban, and Mbeya (p-value of <0.0001 for all comparisons)

^e^A lower proportion of patients in Durban were culture-positive for TB compared to Lusaka and Mbeya (p-values <0.001 and 0.05)

^f^The median TBscore in patients from Mbeya was higher than Cape Town, Lusaka or Durban (p-values <0.0001 for all comparisons)

^g^A greater proportion of patients culture-negative patients received TB treatment empirically in Cape Town compared to Durban (p = 0.039)

### LAM performance

Three (<1 %) LAM strip tests failed on the first attempt and required use of a second strip test to produce valid results. The 3^rd^ LAM strip reader was required in 151/583 (26 %) patients, and in 126/151 (83 %) cases this was for differences in grading bands between the grade 0 and 1 intensity. Thus, excluding grade 0 /1 discordance a 3^rd^ reader was required in only 25/583 (4 %) of patients. Additional file 1 Table S2 compares LAM diagnostic accuracy using the grade-2 versus grade-1 cut-point (pre-January 2014 reference card, Fig. 1A) showing the higher specificity and LR+ of the grade-2 cut-point. Inter-observer agreement as to the presence versus absence of a test band of intensity grade 2 or higher was 97.3 % (kappa 0.87), while agreement regarding the presence versus absence of a test band of intensity grade 1 or higher was 90.9 % (kappa 0.77), p < 0.001. Based on this data, and previously published work [14–16, 26] and in accordance with the updated manufacturer’s reference card (see Additional file 1 Figure S1B), the remaining diagnostic accuracy data presented in the results section is presented using the grade-2 LAM cut-point.

Table 2 shows the diagnostic accuracy of LAM, Xpert MTB/RIF and smear-microscopy amongst HIV-infected patients stratified by CD4 cell count (PPV, NPV, LR+ and LR- shown in Additional file 1 Table S3). Overall, LAM had sensitivity (95 % CI) and specificity (95 % CI) of 22.7 % (16.6–28.7) and 93.0 (90.5–95.6) respectively. LAM specificity did not significantly increase when TB culture-negative patients diagnosed with clinical-TB were considered reference test positive in a secondary analysis [95.4 % (92.1–97.4) versus 93.0 (90.5–95.6), p = 0.2, Additional file 1 Table S4]. LAM specificity was significantly higher at South African compared to Zambian and Tanzanian study sites, both in the primary and secondary analyses [Primary analysis: Cape Town: 99.0 % and Durban: 97.9 % compared to Lusaka: 87.1 % and Mbeya: 89.7 %, p < 0.001 for comparisons between SA sites and Lusaka and p < 0.05 for comparisons between SA sites and Mbeya] (see online Additional file 1 Table S5B and C)]. LAM strip sensitivity (95 % CI) was not significantly higher in CD4 ≤ 100 versus >100cell/mm^3^ [30.4 % (17.1–43.7) versus 18.3 % (12.5–25.9), p = 0.085], while specificity was similar.

**Table 2:** Key diagnostic accuracy measures of LAM (grade 2 cut-point), sputum Xpert MTB/RIF or smear microscopy alone or in combination for culture-confirmed versus culture-negative pulmonary tuberculosis amongst HIV-infected (and refused testing) patients stratified by CD4 cell count

Diagnostic (s)		Sensitivity		Specificity
	n/N	% (95% CI)	n/N	% (95% CI)
LAM alone^a^				
HIV-infected	41/181	22.7 (16.6-28.7)	361/388	93.0 (90.5-95.6)
CD4 ≤ 100 cells/mm^3 b^	14/46	30.4 (17.1-43.7)	70/75	93.3 (87.7-99.0)
CD4 > 100 cells/mm^3^	23/126	18.3 (12.5-25.9)	257/274	93.8 (90.3-96.1)
Xpert MTB/RIF alone				
HIV-infected	70/92	76.1 (67.4-84.8)	182/196	92.9 (88.4-95.7)
CD4 ≤ 100 cells/mm^3^	16/21	76.2 (58.0-94.4)	36/40	90.0 (80.7-99.3)
CD4 > 100 cells/mm^3^	47/63	74.6 (62.7-83.7)	130/138	94.2 (89.0-97.0)
Smear alone				
HIV-infected	39/89	43.8 (34.0-54.2)	179/192	93.2 (88.8-96.0)
CD4 ≤ 100 cells/mm^3^	9/25	36.0 (17.2-54.8)	31/35	88.9 (78.6-99.1)
CD4 > 100 cells/mm^3^	30/63	47.6 (35.8-59.7)	129/136	94.9 (89.8-97.5)
Xpert MTB/RIF and LAM combined^c^				
HIV-infected	72/92	78.3 (69.8-86.7)	169/196	86.2 (81.4-91.0)
CD4 ≤ 100 cells/mm^3^	18/21	85.7 (70.7-100.1)	35/40	87.5 (77.3-97.7)
CD4 > 100 cells/mm^3^	47/63	74.6 (62.7-83.7)	121/138	87.7 (81.2-92.2)
Smear and LAM combined^c^				
HIV-infected	50/89	56.2 (45.9-66.5)	172/192	89.6 (85.3-93.9)
CD4 ≤ 100 cells/mm^3^	11/25	44.0 (24.5-63.5)	28/35	80.0 (66.7-93.3)
CD4 > 100 cells/mm^3^	38/63	60.3 (48.0-71.5)	125/136	91.9 (86.1-95.4)

^a^14/583 HIV-infected and test refused patients had no reference standard result (see Fig. 1) and therefore a total of 569 patients were used for evaluation of urinary LAM performance

^b^48/569 HIV-infected patients with LAM test results missing CD4 cell count data. For no diagnostic accuracy measure did any of the diagnostic tests, either alone or in combination, performed significantly better in CD4 ≤ 100 cells/mm^3^ compared to CD4 > 100 cells/mm^3^ (p > 0.05)

^c^Either test positive is considered as a “positive” result

### LAM testing compared to Xpert MTB/RIF or smear microscopy

The overall sensitivity (95 % CI) of Xpert MTB/RIF (76.1 %, 70/92) was significantly higher than either LAM (22.7 %, 41/181) or smear microscopy (43.8 %, 39/89) (p < 0.001 for both comparisons); similarly in patients with CD4 ≤ 100 cells/mm^3^ Xpert MTB/RIF (76.2 %, 16/21) offered higher sensitivity than either LAM (30.4 %, 14/46, p < 0.001) or smear microscopy (36 %, 9/25, p = 0.006). Xpert MTB/RIF had the highest test LR+ (95 % CI) of 10.7 (9.2–12.4) and correspondingly a PPV (95 % CI) of 83.3 % (74.0–89.8). Overall, but not in patients with CD4 ≤ 100 cells/mm^3^, smear microscopy (43.8 %, 39/89) offered significantly higher sensitivity compared to LAM alone (22.7 %, 41/181, p < 0.001). Similar differences in test sensitivities were seen across all study sites except in Durban where LAM (40 %), sputum Xpert MTB/RIF (60 %) and smear microscopy (50 %) offered similar sensitivities (online Additional file 1 Table S5A). Specificity of LAM (93.0 %), Xpert MTB/RIF (92.9 %) and smear microscopy (93.2 %) used alone was similar irrespective of CD4 strata (p > 0.05 for all comparisons).

### LAM in combination with either sputum-based MTB/RIF or smear microscopy

The diagnostic accuracy of using LAM in combination with either sputum-based Xpert MTB/RIF or smear-microscopy is also shown in Table 2. LAM plus Xpert MTB/RIF (72/92, 78.3 %) did not offer significantly better sensitivity to Xpert MTB/RIF alone (70/92, 76.1 %) (p = 0.7). Similarly, LAM plus smear microscopy (50/89, 56.2 %) did not offer significantly better sensitivity to smear microscopy alone (39/89, 43.8 %) (p = 0.1). In contrast, the specificity of LAM plus Xpert MTB/RIF (169/196, 86.2 %) was significantly lower than either LAM strip (361/388, 93.0 %) or Xpert MTB/RIF (182/189, 92.9 %) testing alone (p = 0.007 versus LAM and p = 0.032 versus Xpert MTB/RIF), and these relationships were unchanged if the analysis was restricted to patients with CD4 ≤ 100 cells/mm^3^. For LAM plus smear microscopy, the overall specificity (172/192, 89.6 %) did not differ significantly compared to either LAM (361/388, 93.0 %) or smear (179/192, 93.2 %) alone (p > 0.05 for both comparisons), however, in patients with CD4 ≤ 100 cells/mm^3^ combined specificity was lower than LAM testing alone (93.0 %) (p = 0.037). The specificity relationships were unchanged in the secondary analysis (Additional file 1 Table S4).

A sensitivity analysis was performed to investigate the impact of the 21 % (28/131) and 27 % (36/130) Non-TB patients in the smear and Xpert study arms respectively that were lost-to-follow up and the 1.5 % (2/131) and 3 % (4/130) that were deceased. No differences in the diagnostic accuracy of LAM alone or with smear/Xpert were noted (data not shown).

### Potential impact on patient-important treatment outcomes of adding point-of-care LAM to sputum-based Xpert MTB/RIF or smear microscopy

**Fig. 2:**
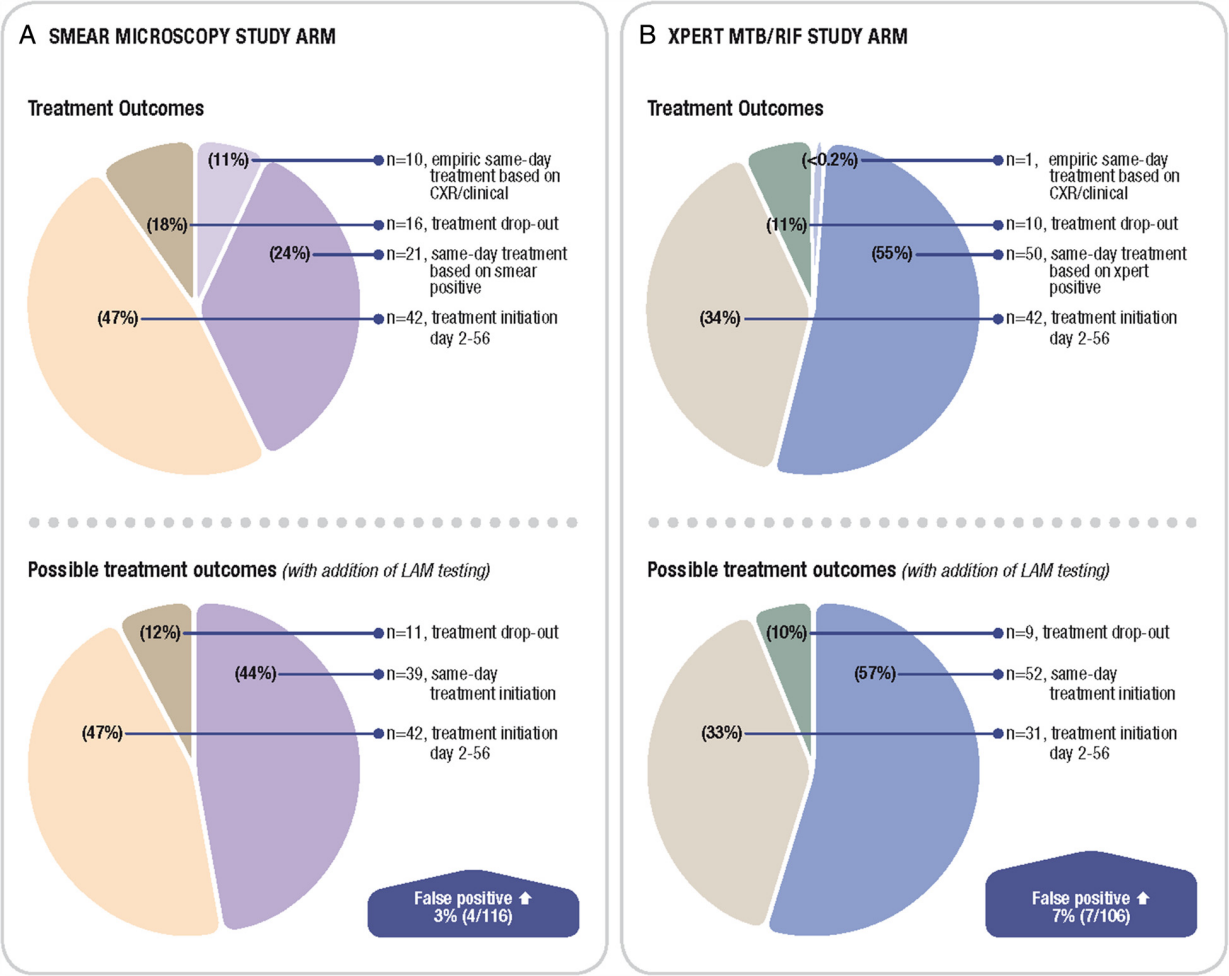
Treatment outcomes in each study arm indicating the potential impact of LAM in TB culture positive patients. **a** Smear-microscopy **b** clinic-based Xpert MTB/RIF testing. ‘False-positives’: These indicate patients that were sputum culture-negative, smear or Xpert MTB/RIF-negative and not given empiric TB treatment by the attending clinician that would have been LAM positive. These patients would have thus received inappropriate anti-TB treatment

In the parent study, the reason for treatment initiation, time-to-treatment and failure to initiate treatment in culture-positive TB patients (‘dropout’) was recorded (Fig. 2). The potential impact on these outcomes of adding point-of-care LAM (in the manner described in methods section) is also shown in Fig. 2. In the smear microscopy study arm with the use of empiric treatment based on CXR, use of POC LAM would neither have significantly decreased dropout [18 % (16/89) to 12 % (11/89), p = 0.3], nor significantly increased same-day treatment initiation [35 % (31/89) to 44 % (39/89), p = 0.2]. However, in primary care settings without same-day CXR facilities, POC LAM would have significantly increased same-day treatment initiation [24 % (21/89) to 44 % (39/89, p = 0.004]. Potentially unnecessary treatment initiation would have increased by 3 % (4/116) due to LAM ‘false-positives’. In the Xpert MTB/RIF study arm, LAM would neither have significantly decreased treatment dropout [11 % (10/92) to 10 % (9/92), p = 0.8] nor significantly increased same-day treatment initiation [55 % (51/92) to 57 % (52/92), p = 0.9]. However, potentially unnecessary treatment initiation would have increased by 7 % (7/106) due to LAM ‘false-positives’.

### LAM as a potential prognostic marker

Table 3 shows all cause 6-month mortality, lost-to-follow-up and TBscore at enrolment amongst HIV-infected/status unknown patients stratified by LAM result. LAM strip positive versus negative patients had: i) higher all cause 6-month mortality [25 % versus 11 %, P = 0.02], ii) higher lost-to-follow-up [54 % versus 30 %, p < 0.001], and iii) a higher baseline TB-related morbidity score [7 (6–8) versus 5 (4–7), p = 0.03]. However, in the multivariate analyses shown in Table 4, LAM positivity was only a predictor of the combined outcome of lost-to-follow-up/death but not 6-month mortality or baseline TBscore. In addition, TB-related morbidity outcomes, as measured by the amount of improvement from enrolment in TB-score at 2- and 6-months post-initiation of effective anti-TB treatment were similar amongst LAM strip positive versus negative patients [4(3–5) versus 4 (2.5–5), p = 0.136] (online Additional file 1 Table S6).

**Table 3:** Morbidity (measured by TBscore), 6-month all cause mortality and lost-to-follow up stratified by baseline results of LAM in HIV-infected (or test refused) patients^**a**^

	All HIV-infected n/N (%)	LAM strip positive n/N (%)	LAM strip negative n/N (%)	P-value
6-month mortality (N = 393)^**b**^	49/393 (13)	9/32 (25)	40/361 (11)	0.021
TB culture-positive only (N = 123)^b^		6/17 (35)	15/106 (14)	0.032
Lost-to-follow up	190/583 (33)	38 (54)	152 (30)	<0.001
Culture-positive (*N* = 58)		24/41 (59)	34/140 (24)	<0.001
TBscore at enrolment				
Culture-positive (N = 181)	6 (4-7.5)	7 (6-8)	5 (4-7)	0.025

^a^14 HIV-infected and test refused patients with no reference standard result (see Fig. 1) excluded from the analyses of index test diagnostic accuracy are included in this analysis of morbidity and mortality. Thus a total of 583 patients are included in this table’s analysis

^b^Of the 583 HIV-infected patients with a LAM result, 190 were lost-to-follow-up at 6-months. Of the 393 patients with complete 6-month follow-up data, 123 were culture-positive TB

**Table 4:** Multivariate analysis showing predictors of all-cause mortality, lost-to-follow-up/mortality and baseline TB-related morbidity in HIV-infected patients overall and restricted to culture-positive TB patients only^**a**^

	*P*-value	AOR	Lower 95 % CI	Upper 95 % CI
6-month all-cause mortality				
All HIV-infected (*n* ^*^ = 173)				
Age, years	0.051	0.934	0.867	0.997
CD4 cell count, cells/mm^3^	0.038	0.996	0.991	0.999
TB culture positive (pos/neg)	0.007	9.207	2.069	53.4
Baseline TB score	0.050	1.349	1.012	1.86
LAM strip result^b^	0.170	2.762	0.613	11.72
**TB culture positive (** ***n*** **= 117)**				
CD4 cell count, cells/mm^3^	0.069	0.9951	0.989	0.999
Baseline TB score	0.007	1.683	1.19	2.555
LAM strip result	0.252	2.59	0.495	13.72
Lost-to-follow-up or deceased				
All HIV-infected (*n* = 268)				
Age, years	0.048	0.966	0.932	0.999
Zambia	<0.0001	12.52	4.077	45.15
Durban, South Africa	0.052	3.701	1.027	14.82
CD4 cell count, cells/mm^3^	0.058	0.998	0.997	1
LAM strip result	**0.002**	**4.382**	**1.834**	**11.58**
TB culture positive (*n* = 170)				
Age, years	0.010	0.944	0.903	0.985
Zambia	0.002	5.897	2.055	18.61
Baseline TB score	0.006	1.301	1.085	1.583
LAM strip result	**0.007**	**4.717**	**1.638**	**15.85**
Baseline TB score (*n* = 146)				
TB culture positive				
CD4 cell count, cells/mm^3^	0.02716	0.9977	0.9958	0.9997
LAM strip result (pos/neg)	0.1771	1.886	0.7542	4.717

Abbreviations: *ARV*, anti-retroviral; *CI*, confidence interval; *AOR*, adjusted odds ratio

^**a**^Additional co-variates included all multivariate models as potential confounders include: study arm (Xpert MTB/RIF or smear), study site, sex, age. Additional co-variates evaluated as additional predictors of outcomes included: positive initial TB result (Xpert MTB/RIF or smear), ARV treatment status, previous TB and CXR suggestive of TB. LAM strip test is always shown while only significant co-variates in each multivariate regression are shown

^*^n = number included in each of the multivariate models depending on outcome and sub-group considered

^b^LAM strip results are considered as a binary variable (positive/negative) using the grade 2 cut-point. Results of the multivariate analysis do not change if LAM strip results are considered as continuous variable from grade 0-5

## Discussion

In this multi-centre out-patient study of patients with suspected PTB able to provide expectorated sputum for diagnostic testing, LAM had poor overall sensitivity, which did not significantly improve in patients with CD4 cell counts less than 100 cells/ml. LAM was not able to improve on the diagnosis offered by either sputum-based Xpert MTB/RIF or smear microscopy alone. Due to this lack of incremental utility over sputum-based tools, LAM seems unlikely to be able to improve outpatient important treatment outcomes. Indeed, although LAM could offer some prognostic utility in identifying patients at high risk of death or loss-to-follow-up, there was no impact on patient dropout or morbidity. Adjunctive LAM testing may only increase same-day treatment initiation in clinic settings using sputum smear microscopy where same-day chest radiography is not available to guide empiric same-day treatment decisions. It may be argued that our study only considered outpatients able to expectorate sputum, and LAM may offer important incremental value for patients unable to provide sputum for diagnostic testing. However, this study’s patient population is the major subgroup presenting to primary care facilities for frontline testing and although preliminary data suggest that this is not the subgroup most likely to benefit from LAM, data is nevertheless essential for clear recommendations to guide implementation across TB programs. Indeed, program directors continue to question why the only commercially available and affordable POC TB test is unavailable for frontline testing? The data outlined here offers valuable insights.

Published data, albeit limited, indicate that LAM sensitivity is increased with higher circulating LAM levels, occurring with higher mycobacterial disease burden, extrapulmonary TB, lower CD4 cell count and WHO clinical stage 3 and 4 in out- and in-patient settings [11, 27–34]. Moreover, LAM and sputum smear microscopy identified non-overlapping sub-groups of culture-positive TB, thereby offering additive diagnostic value [11–14, 16, 35]. By contrast, we found no incremental benefit of LAM. There are a number of possible explanations. Firstly, sputum-scarce TB, smear-negative TB, and EPTB is more common in hospital-based and pre-ART screening cohorts and thus smear microscopy sensitivity is more likely to be reduced, which in turn would increase the incremental benefit offered by LAM testing. Secondly, differences in sputum smear microscopy staining and concentration methodologies across different studies affect smear microscopy sensitivity [13]. Thirdly, in contrast to other LAM-based studies [11, 12, 14] our study did not offer sputum induction to improve sputum sampling and thus, those unable to spontaneously produce two spot sputa were excluded. Sputum induction was not offered as the pragmatic study design of the parent study reflects the reality that sputum induction facilities remain unavailable in the majority of routine primary care clinic settings. Nevertheless, the inability of LAM testing to improve the diagnostic yield of a single sputum-based Xpert MTB/RIF, irrespective of declining CD4 cell count, is consistent with the findings of Lawn *et al.* and reflect the superior sensitivity of sputum-based Xpert MTB/RIF for the diagnosis of pulmonary TB [11, 36, 37].

In our initial study of LAM amongst hospitalised HIV-infected patients with advanced immunosuppression we noted that test specificity and inter-reader agreement was optimised (>95 % for both) by use of an alternative grade-2 rather than the manufacturer’s initially suggested grade-1 cut-point [16]. Based on these findings, the manufacturer’s reference card has been updated as of January 2014 so that the first positive visual band corresponds to the grade-2 intensity band of the old reference card (see Fig. 1 of the online supplement, www.alere.com).

Independent of cut-point selection, suboptimal LAM specificity (<95 % as recommended by an expert committee for point-of-care TB testing [38]), particularly in countries north of South Africa, remains a concern. Indeed we noted lower test specificity in Tanzania and Zambia compared to the two South African study sites. Reasons for this may include different degrees of the unavoidable misclassification bias associated with a single sputum culture to correctly classify TB in HIV-infected patients with advancing immunosuppression. However, in our secondary analysis excluding ‘probable or clinical TB’ the specificity in Zambia and Mbeya remained lower. Kroidl et al. found cross-contamination of the LAM ELISA from dust, soil and stool in Tanzania, and we have demonstrated cross-reacting LAM-like glycolipid antigens in *Nocardia* and *Candida spp*. [30, 39]. Sterile collection of urine samples is essential, especially in countries north of South Africa, and in a recent Ugandan in- and out-patient study LAM specificity was 95 % [14].

LAM offered limited incremental diagnostic benefit over sputum-based diagnostics. In contrast to studies showing incremental benefit of LAM in hospitalised patients with sputum-scarce TB and EPTB [14, 16] or those identifying patients with TB missed by empiric treatment initiation but identified by LAM [40]. Consequently, our study suggests LAM would have minimal potential impact on patient important treatment outcomes. In fact, test specificity was significantly lower when combining Xpert MTB/RIF with LAM for both a culture and composite reference standard with the potential to increase inappropriate treatment initiation. Thus, sputum-based diagnosis, especially where Xpert MTB/RIF is available, should be preferred in HIV-infected outpatients able to spontaneously provide sputa. However, LAM may potentially improve same-day treatment initiation in the clinic setting where only sputum smear microscopy is performed and no chest radiography facilities are available. In addition, LAM may still offer i) important added diagnostic benefit where the performance of sputum-based tests is reduced such as sputum-scarce TB, extrapulmonary TB, mycobacteremia [27], and/ or renal TB [41], and ii) important prognostic and treatment monitoring utility [18].

This study had several limitations and strengths. It is the first large multicentre study in primary care practice, allowing for accurate evaluation of diagnostic accuracy across three sub-Saharan African countries. The design of the parent study offered a unique opportunity to evaluate the diagnostic accuracy of LAM in a well-defined out-patient population able to provide sputum for diagnostic testing and hence, to estimate the potential impact of LAM when combined with either Xpert MTB/RIF or smear in this patient group. Misclassification bias was a potential problem in our study as a single sputum culture can miss TB cases amongst HIV-infected patients. However, in an alternative analysis where TB culture-negative patients diagnosed as clinical TB and initiating treatment are considered the reference test, no significant difference in LAM specificity was noted and study conclusions were unaltered. In addition, a sensitivity analysis with lost-to-follow-up and deceased patients excluded from the non-TB group i.e. considered unclassifiable, did not significantly alter diagnostic accuracy measures. LAM was performed on frozen rather than fresh samples which could have reduced test sensitivity, however, meta-analysis data suggests no differences in diagnostic accuracy using frozen rather than fresh samples [42]. Consequently, impact data for adjunctive LAM is extrapolated. The study was not powered to detect small differences in sub-groups (CD4 strata, treatment dropouts) and thus small incremental benefits of LAM in these sub-groups may not have been detected. Likewise, the small number of study deaths limited power to examine LAM as a predictor of mortality in the multivariate analysis.

In conclusion, LAM strip testing had poor sensitivity amongst HIV-infected outpatients able to provide expectorated sputum for diagnostic testing. There was no incremental diagnostic benefit over either Xpert MTB/RIF or smear microscopy. If used as an adjunctive diagnostic tool in this setting, it is unlikely to impact patient-important treatment outcomes such as morbidity, patient dropout, or same-day treatment initiation, except in smear microscopy only clinics with no chest radiography. Potential gains need to be weighed against the likely increase in inappropriate ‘false-positive’ treatment. However, further impact-orientated studies focused on mortality and morbidity benefits in ill hospitalised patients are warranted.

## Additional file

Additional file 1: Figure S1A. Pre-January 2014 LAM strip test manufacturer’s reference card illustrating visual intensity grades 0-5. Figure S1B: January 2014 new LAM strip test manufacturer’s reference card illustrating visual intensity grades 0-4. Table S1. Variables used to calculate the TB score as defined by Wejse *et al.* (2008)^1^. Each patient was scored at baseline, 2 months and 6 months. Table S2. Comparative diagnostic accuracy of two LAM strip test grade cut-points (old reference card) in HIV-infected patients and stratified by CD4 cell count. Table S3. Additional diagnostic accuracy measures (Likelihood ratios and predictive values) of LAM (grade 2 cut-point), sputum Xpert MTB/RIF or smear microscopy alone or in combination for culture-confirmed versus culture-negative pulmonary tuberculosis amongst HIV-infected (and refused testing) patients stratified by CD4 cell count (TB prevalence = 31 %). Table S4. Diagnostic accuracy of LAM (grade 2 cut-point) alone or in combination with either Xpert MTB/RIF or smear microscopy for culture-positive and clinical versus culture-negative pulmonary tuberculosis amongst HIV-infected (and refused testing) patients and stratified by CD4 cell count. Table S5A. Sensitivity of different diagnostic tests alone and in combination in HIV-infected (and refused testing) patients with culture-positive tuberculosis, stratified by study site. Table S5B. Specificity of different diagnostic tests alone or in combination in all patients culture-negative for tuberculosis, stratified by study site. Table S5C. Specificity of different diagnostic tests alone or in combination in HIV-infected patients culture-negative for tuberculosis and without clinical TB^¶^ stratified by study site. Table S6. Changes in 2-month and 6-month TB-related morbidity indices in patients treated for TB according to baseline culture status, stratified by LAM result.

## Abbreviations

LAM: Lipoarabinomannan. In manuscript used as abbreviation for Determine^TM^ TB LAM Ag lateral flow strip test (Alere, USA)
HIV: Human immunodeficiency virus
TB: Tuberculosis
EPTB: Extra-pulmonary tuberculosis
Xpert MTB/RIF: GeneXpert MTB/RIF assay (Cepheid, USA)
POC: Point-of-care
DOTS: Directly observed Treatment Short-course
WHO: World Health Organisation
RCT: Randomised controlled trial
MGIT: Microscopic growth in-tube
NaOH: Sodium hydroxide
ZN: Ziehl-Neelsen
CD4: CD4 T-cell count
LR+: Positive likelihood ratio
PPV: Positive predictive value
LR-: Negative likelihood ratio
NPV: Negative predictive value
ELISA: Enzyme linked immunosorbent assay
