# Supplementary material for: Test characteristics and potential impact of the urine LAM lateral flow assay in HIV-infected outpatients under investigation for TB and able to self-expectorate sputum for diagnostic testing
Source: BMC Infect Dis. 2015 Jul 9;15:262. doi: 10.1186/s12879-015-0967-z (PMC4495934; doi:10.1186/s12879-015-0967-z)
Supplement: Additional file 1: Figure S1A. — Pre-January 2014 LAM strip test manufacturer’s reference card illustrating visual intensity grades 0-5. Figure S1B: January 2014 new LAM strip test manufacturer’s reference card illustrating visual intensity grades 0-4. Table S1. Variables used to calculate the TB score as defined by Wejse et al. (2008)1. Each patient was scored at baseline, 2 months and 6 months. Table S2. Comparative diagnostic accuracy of two LAM strip test grade cut-points (old reference card) in HIV-infected patients and stratified by CD4 cell count. Table S3. Additional diagnostic accuracy measures (Likelihood ratios and predictive values) of LAM (grade 2 cut-point), sputum Xpert MTB/RIF or smear microscopy alone or in combination for culture-confirmed versus culture-negative pulmonary tuberculosis amongst HIV-infected (and refused testing) patients stratified by CD4 cell count (TB prevalence = 31 %). Table S4. Diagnostic accuracy of LAM (grade 2 cut-point) alone or in combination with either Xpert MTB/RIF or smear microscopy for culture-positive and clinical versus culture-negative pulmonary tuberculosis amongst HIV-infected (and refused testing) patients and stratified by CD4 cell count. Table S5A. Sensitivity of different diagnostic tests alone and in combination in HIV-infected (and refused testing) patients with culture-positive tuberculosis, stratified by study site. Table S5B. Specificity of different diagnostic tests alone or in combination in all patients culture-negative for tuberculosis, stratified by study site. Table S5C. Specificity of different diagnostic tests alone or in combination in HIV-infected patients culture-negative for tuberculosis and without clinical TB¶ stratified by study site. Table S6. Changes in 2-month and 6-month TB-related morbidity indices in patients treated for TB according to baseline culture status, stratified by LAM result. [file 12879_2015_967_MOESM1_ESM.doc]

**Supplementary information**

**Methods**

***Symptom eligibility information***

Individuals infected with HIV (both ART-naïve and -treated patients), or whose HIV status was unknown, were considered symptomatic if a cough, night sweats, fever, or weight loss were present. Patients who did not meet these criteria or who were unwilling or unable to give informed consent were excluded

***Final diagnostic categorisation***

The reference standard for the primary analysis of diagnostic accuracy was a single sputum liquid culture for *Mycobacterium tuberculosis*. Given the limitations of this reference standard in HIV-infected patients with advanced immunosuppression an alternative analysis of diagnostic accuracy is performed with Clinical TB patients considered as TB positive. The following diagnostic categories were used for this analysis:

i) Definite TB if the sputum collected at recruitment grew acid-fast bacilli identified as *Mycobacterium tuberculosis* complex.

ii) Clinical TB if culture-negative for TB but initiated on anti-tuberculous treatment (at any time during the 6-month study period) based on either a sputum-positive Xpert MTB/RIF or smear microscopy result, a chest x-ray suggestive of TB or clinical suspicion alone, and

iii) Non TB if culture-negative, and Xpert MTB/RIF or sputum smear-microscopy negative and not initiated on TB treatment.

***LAM strip test methodology***

All patients were required to give a spot urine sample (10-30ml) collected in a sterile container at enrollment. Urine was stored at -20°C for later batched testing. The LAM strip test was performed on unprocessed urine according to manufacturer’s instructions. Briefly, urine was thawed, mixed and 60μl pipetted onto the lateral flow strip loading bay. After 25 minutes, two readers in ambient laboratory lighting conditions, and blinded to the clinical patient details and clinical TB status, independently evaluated the LAM strips for all study patients via the following procedure: after confirming test validity by identifying the presence of a band in the positive control window, the intensity of the colour band (if any) in the patient window was read by comparison with the manufacturer-provided visual reference scale card (graded 0 – 5 depending on band intensity). Using the manufacturer-recommended grade 1 cut-point, a band of visual intensity ≥ grade 1 in the patient window was classified test ‘positive’ while only the complete absence of a band (grade 0) in the patient window was classified test ‘negative’. Accuracy was assessed at various alternative cut-points to select one for optimal rule-in value. For example, if the grade 2 cut-point was selected the complete absence of band (grade 0) as well as a faint band (grade 1) was classified test ‘negative’ and only a band of visual intensity ≥ grade 2 was considered test ‘positive’. The test was reported as indeterminate if a broken/ incomplete band was seen in the patient window. A test was reported as failed if no control band was identified. Each reader graded the LAM strips blinded to the results of the other. The results were then compared and if discrepant, a third reader was asked to independently grade the strip and the consensus result was used.

**Figure S1A**: Pre-January 2014 LAM strip test manufacturer’s reference card illustrating visual intensity grades 0-5

**Figure S1B:** January 2014 new LAM strip test manufacturer’s reference card illustrating visual intensity grades 0-4


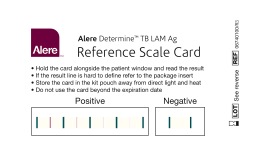


**In the updated and currently available reference card the first positive band corresponds to the grade-2 intensity band in the old, pre-January 2014 reference card.**

**Table S1.** Variables used to calculate the TB score as defined by Wejse *et al.* (2008)[^1^](#_ENREF_1). Each patient was scored at baseline, 2 months and 6 months.

| **Parameters** | **Points assigned** (Maximum score is 13) |
| --- | --- |
| Self-reported | |
| Cough | 1 |
| Haemoptysis | 1 |
| Dyspnoea | 1 |
| Chest pain | 1 |
| Night sweats | 1 |
| Clinically examined | |
| Anaemic conjunctivae | 1 |
| Tachycardia | 1 |
| Positive finding at lung auscultation | 1 |
| Axillary temperature > 37.0 °C | 1 |
| BMI < 18.0 | 1 |
| BMI < 16.0 | 1 |
| MUAC < 220 mm | 1 |
| MUAC < 200 mm | 1 |

Abbreviations: BMI, body mass index; MUAC, middle upper arm circumference.

**Table S2. Comparative diagnostic accuracy of two LAM strip test grade cut-points (old reference card) in HIV-infected patients and stratified by CD4 cell count**

| **Patient group** | **HIV-infected** | | **CD4≤200 cells/mm^3 §^** | | **CD4≤100 cells/mm^3 §^** | | **P-value**^†^ |
| --- | --- | --- | --- | --- | --- | --- | --- |
|  | **Grade 1 cut-point** | **Grade 2 cut-point** | **Grade 1 cut-point** | **Grade 2 cut-point** | **Grade 1 cut-point** | **Grade 2 cut-point** |  |
| **Sensitivity (95% CI) n/N** | 37.6 (30.5-44.6)^*^ 68/181 | 22.7 (16.6-28.7)^*^ 41/181 | 39.2 (29.5-48.9)^*1^ 38/97 | 23.7 (15.2-32.2)^*1^ 23/97 | 50 (35.5-64.4) 23/46 | 30.4 (17.1-43.7) 14/46 | ^*^p=0.002 ^*1^p=0.02 |
| **Specificity (95% CI)** | 79.1 (75.1-83.2)^*^ 307/388 | 93.0 (90.5-95.6)^*^ 361/388 | 80.3 (73.9-86.6)^*1^ 122/152 | 94.1 (90.3-97.8)^*1^ 143/152 | 76.0 (66.3-85.7)^*2^ 57/75 | 93.3 (87.7-99.0)^*2^ 70/75 | ^*^p<0.0001 ^*1^p=0.0003 ^*2^p=0.003 |
| **LR Positive (95% CI)** | 1.8 (1.7-1.9) | 3.3 (2.6-4.1) | 2.0 (1.7-2.3) | 4.0 (2.4-6.6) | 2.1 (1.7-2.5) | 4.6 (2.2-9.3) | n/c |
| **LR Negative (95% CI)** | 0.79 (0.77-0.80) | 0.83 (0.82-0.84) | 0.76 (0.73-0.79) | 0.81 (0.79-0.83) | 0.66 (0.60-0.72) | 0.75 (0.70-0.79) | n/c |
| **Positive PV (95% CI) n/N** | 45.6 (37.9-53.6)^*^ 68/149 | 60.3 (68.0-75.8)^*^ 41/68 | 55.9 (44-1-67.1) 38/68 | 71.9 (54.6-84.4) 23/32 | 56.1 (41.0-70.1) 23/41 | 73.7 (51.2-88.2) 14/19 | ^*^p=0.045 |
| **Negative PV (95% CI) n/N** | 73.1 (68.7-77.1) 307/420 | 72.1 (68.0-75.8) 361/501 | 67.4 (60.3-73.8) 122/181 | 65.9 (59.4-71.9) 143/217 | 71.3 (60.5-80.0) 57/80 | 68.6 (59.1-76.8) 70/102 | n/s |

^§^48/569 HIV-infected patients with LAM test results missing CD4 cell count data.

^†^P-values shown are between LAM cut-points for each diagnostic accuracy measure and are marked with an asterisk (^*^ or ^*1^ or ^*2^) to indicate comparison. If no p-value is shown then no significant difference was found (p>0.05)

**Table S3. Additional diagnostic accuracy measures (Likelihood ratios and predictive values) of LAM (grade 2 cut-point), sputum Xpert MTB/RIF or smear microscopy alone or in combination for culture-confirmed versus culture-negative pulmonary tuberculosis amongst HIV-infected (and refused testing) patients stratified by CD4 cell count (TB prevalence=31%).**

| **Diagnostic(s)** | **LR Positive** | **LR Negative** | **Positive PV** | **Negative PV** |
| --- | --- | --- | --- | --- |
|  | **(95% CI)** | **(95% CI)** | **(95% CI)** | **(95% CI)** |
| **LAM alone^‡^** | | | | |
| HIV-infected | 3.3 (2.6-4.1) | 0.83 (0.82-0.84) | 60.3 (48.4-71.1) | 72.1 (68.0-75.8) |
| CD4≤100 cells/mm^3 §^ | 4.6 (2.2-9.3) | 0.75 (0.70-0.79) | 73.7 (51.2-88.2) | 68.6 (59.1-76.8) |
| CD4>100 cells/mm^3^ | 2.9 (1.8-4.8) | 0.87 (0.85-0.89) | 57.5 (42.2-71.5) | 71.4 (66.5-75.8) |
| **Xpert MTB/RIF alone** | | | | |
| HIV-infected | 10.7 (9.2-12.4) | 0.26 (0.24-0.28) | 83.3 (74.0-89.8) | 89.2 (84.2-92.8) |
| CD4≤100 cells/mm^3^ | 7.6 (4.5-12.9) | 0.26 (0.18-0.39) | 80 (58.4-91.9) | 87.8 (74.5-94.7) |
| CD4>100 cells/mm^3^ | 12.9 (9.9-16.7) | 0.27 (0.24-0.31) | 85.5 (73.8-92.4) | 89.0 (82.9-93.1) |
| **Smear alone** | | | | |
| HIV-infected | 6.4 (5.2-8.0) | 0.60 (0.58-0.63) | 75.0 (61.8-84.8) | 78.2 (72.4-83.0) |
| CD4≤100 cells/mm^3^ | 3.2 (1.3-7.6) | 0.72 (0.63-0.82) | 69.2 (42.4-87.3) | 66.0 (54.1-77.3) |
| CD4>100 cells/mm^3^ | 9.3 (6.5-13.2) | 0.55 (0.52-0.59) | 81.1 (65.8-90.5) | 79.6 (72.8-85.1) |
| **Xpert MTB/RIF and LAM combined^†^** | | | | |
| HIV-infected | 5.7 (5.2-6.2) | 0.25 (0.23-0.28) | 72.7 (63.2-80.5) | 89.4 (84.2-93.0) |
| CD4≤100 cells/mm^3^ | 6.9 (4.6-10.3) | 0.16 (0.08-0.31) | 78.3 (58.1-90.3) | 92.1 (79.2-97.3) |
| CD4>100 cells/mm^3^ | 6.1 (5.3-6.8) | 0.29 (0.26-0.33) | 73.4 (61.5-82.7) | 88.3 (81.9-92.7) |
| **Smear and LAM combined^†^** | | | | |
| HIV-infected | 5.4 (4.7-6.1) | 0.49 (0.46-0.52) | 71.4 (60.0-80.7) | 81.5 (75.7-86.2) |
| CD4≤100 cells/mm^3^ | 2.2 (1.3-3.7) | 0.70 (0.60-0.82) | 61.1 (38.6-79.7) | 66.7 (51.6-79.0) |
| CD4>100 cells/mm^3^ | 7.5 (6.0-9.2) | 0.43 (0.40-0.47) | 77.6 (64.1-87.0) | 83.3 (76.6-88.5) |

**^‡^14/583 HIV-infected and test refused patients had no reference standard result (see Figure 1) and therefore a total of 569 patients were used for evaluation of urinary LAM performance**

^§^48/569 HIV-infected patients with LAM test results missing CD4 cell count data. For no diagnostic accuracy measure did any of the diagnostic tests, either alone or in combination, performed significantly better in CD4≤100 cells/mm^3^ compared to CD4>100 cells/mm^3^ (p>0.05).

^†^Either test positive is considered as a “positive” result

**Table S4.** Diagnostic accuracy of LAM (grade 2 cut-point) alone or in combination with either Xpert MTB/RIF or smear microscopy for culture-positive and clinical versus culture-negative pulmonary tuberculosis amongst HIV-infected (and refused testing) patients and stratified by CD4 cell count^¶^.

|  | **Sensitivity** | | **Specificity** | | **LR Positive** | **LR Negative** | **Positive PV** | **Negative PV** |
| --- | --- | --- | --- | --- | --- | --- | --- | --- |
|  | **n/N** | **% (95% CI)** | **n/N** | **% (95% CI)** | **(95% CI)** | **(95% CI)** | **(95% CI)** | **(95% CI)** |
| **LAM alone**^‡^ | | | | | | | | |
| HIV-infected | 56/308 | 18.2 (14.3-22.9) | 249/261 | 95.4 (92.1-97.4) | 4.0 (2.9-5.5) | 0.86 (0.85-0.87) | 82.4 (71.7-89.6) | 49.7 (45.3-54.1) |
| CD4≤100 cells/mm^3 §^ | 16/74 | 21.6 (13.8-32.3) | 44/47 | 93.6 (82.8-97.8) | 3.4 (1.1-10.2) | 0.84 (0.80-0.87) | 84.2 (62.4-94.5) | 43.1 (34.0-52.8) |
| CD4>100 cells/mm^3^ | 34/214 | 15.9 (11.6-21.4) | 180/186 | 96.8 (93.1-98.5) | 4.9 (2.6-9.3) | 0.87 (0.86-0.88) | 85.0 (70.9-92.9) | 50.0 (44.9-55.1) |
| **Xpert MTB/RIF alone** | | | | | | | | |
| HIV-infected | 84/157 | 53.5 (45.7-61.1) | 131/131 | 100 (97.2-100) | N/C | 0.47 (0.45-0.48) | 100 (95.6-100) | 64.2 (57.4-70.5) |
| CD4≤100 cells/mm^3^ | 20/36 | 55.6 (39.6-70.5) | 25/25 | 100 (86.7-100) | N/C | 0.44 (0.39-0.50) | 100 (83.9-100) | 61.0 (45.7-74.3) |
| CD4>100 cells/mm^3^ | 56/107 | 52.3 (43.0-61.6) | 94/94 | 100 (96.1-100) | N/C | 0.48 (0.46-0.50) | 100 (93.6-100) | 64.8 (56.8-72.1) |
| **Smear alone** | | | | | | | | |
| HIV-infected | 50/151 | 33.1 (26.1-41.0) | 128/130 | 98.5 (94.6-99.6) | 21.5 (7.5-62.1) | 0.68 (0.67-0.69) | 96.2 (87.0-98.9) | 55.9 (49.4-62.2) |
| CD4≤100 cells/mm^3^ | 12/38 | 31.6 (19.1-47.5) | 21/22 | 95.5 (78.2-99.2) | 6.9 (0.7-70.3) | 0.72 (0.66-0.78) | 92.3 (66.7-98.6) | 44.7 (31.4-58.8) |
| CD4>100 cells/mm^3^ | 36/107 | 33.6 (25.4-43.0) | 91/92 | 98.9 (94.1-99.8) | 31.0 (3.9-244.7) | 0.67 (0.65-0.69) | 97.3 (86.2-99.5) | 56.2 (48.5-63.6) |
| **Xpert MTB/RIF and LAM combined^†^** | | | | | | | | |
| HIV-infected | 91/157 | 58.0 (50.1-65.4) | 123/131 | 93.9 (8.4-96.9) | 9.5 (7.3-12.3) | 0.45 (0.43-0.46) | 91.9 (84.9-95.9) | 65.1 (58.0-71.5) |
| CD4≤100 cells/mm^3^ | 22/36 | 61.1 (44.9-75.2) | 24/25 | 96 (80.5-99.3) | 15.3 (2.0-114.8) | 0.41 (0.35-0.47) | 95.7 (79.0-99.2) | 63.2 (47.3-76.6) |
| CD4>100 cells/mm^3^ | 60/107 | 56.1 (46.6-65.1) | 90/94 | 95.7 (89.6-98.3) | 13.2 (7.9-22.1) | 0.46 (0.44-0.48) | 93.8 (85.0-97.5) | 65.7 (57.4-73.1) |
| **Smear and LAM combined^†^** | | | | | | | | |
| HIV-infected | 64/151 | 42.4 (34.8-50.4) | 124/130 | 95.4 (90.3-97.9) | 9.2 (6.4-13.3) | 0.60 (0.59-0.62) | 91.4 (82.5-96.0) | 58.8 (52.0-65.2) |
| CD4≤100 cells/mm^3^ | 15/38 | 39.5 (25.6-55.3) | 19/22 | 86.4 (66.7-95.3) | 2.9 (1.2-6.8) | 0.70 (0.63-0.78) | 83.3 (60.8-94.2) | 45.2 (31.2-60.1) |
| CD4>100 cells/mm^3^ | 44/107 | 41.1 (32.3-50.6) | 89/92 | 96.7 (90.9-98.9) | 12.6 (6.2-25.8) | 0.61 (0.59-0.63) | 93.6 (90.1-98.9) | 58.6 (60.6-66.1) |

**^‡^14/583 HIV-infected and test refused patients had no reference standard result (see Figure 1) and therefore a total of 569 patients were used for evaluation of urinary LAM performance**

^¶^Reference standard “positive” includes sputum TB culture-positive patients together with those diagnosed with clinical TB (N=127). Clinical TB includes all culture-negative patients given anti-tuberculosis treatment based on either a sputum-positive Xpert MTB/RIF (n=14), smear microscopy (n=11), Chest x-ray suggestive of TB (n=96) or clinical suspicion alone (n=6).

**^§^48/569 HIV-infected patients with LAM test results missing CD4 cell count data.**

^†^Either test positive is considered as a “positive” result.

**Table S5A.**  Sensitivity of different diagnostic tests alone and in combination in HIV-infected (and refused testing) patients with culture-positive tuberculosis, stratified by study site.

| **Site-specific performance (HIV-infected only)** | **Sensitivity (95% Confidence interval) of diagnostic test or test combination, n/N^*^** | | | | | |
| --- | --- | --- | --- | --- | --- | --- |
|  | **LAM** | **MTB/RIF** | **MTB/RIF + LAM** | **Smear** | **Smear + LAM** | **P-value^†^** |
| **Cape Town** | 5.1 (0.0-12.1)^**1^ 2/39 | 70.0 (50.0-90.1)^*^ 14/20 | 70.0 (50.0-90.1) 14/20 | 47.4 (24.9-69.8) ^*1^ 9/19 | 47.4 (24.9-69.8) 9/19 | ^*^p<0.001 ^*1^p<0.001 |
| **Durban** | 40.0 (20.8-59.2) 10/25 | 60.0 (35.3-84.8) 9/15 | 66.7 (42.8-90.5) 10/15 | 50.0 (19.0-81.0) 5/10 | 70.0 (41.6-98.4) 7/10 | n/s |
| **Lusaka** | 27.0 (18.3-35.7)^*^ 27/100 | 83.7 (73.3-94.0)^* *1^ 41/49 | 85.7 (75.9-95.5) 42/49 | 41.2 (27.7-54.7)^*^ 21/51 | 56.9 (43.3-70.5)^*1^ 29/51 | ^*^p<0.001 ^*1^p=0.003 |
| **Mbeya** | 11.8 (0.0-27.1)^*^ 2/17 | 75.0 (45.0-100.0)^*^ 6/8 | 75.0 (45.0-100.0) 6/8 | 44.4 (12.0-76.9) 4/9 | 55.6 (23.1-88.0) 5/9 | ^*^p=0.002 |

^*^Denominator is the number of TB culture positive patients in the indicated group. Parent study [^2^](#_ENREF_2) involved randomisation to either Xpert MTB/RIF or smear-microscopy testing hence reduction in numbers.

^†^P-values shown are between diagnostic test (or test combinations) for each patient group (rows) and are marked with an asterisk (^*^ or ^*1^) to indicate tests compared. If no p-value is shown then no significant difference is present (p>0.05)

**Table S5B.** Specificity of different diagnostic tests alone or in combination in all patients culture-negative for tuberculosis, stratified by study site.

| **Site-specific performance (HIV-infected only)** | **Specificity (95% Confidence interval) of diagnostic test or test combination, n/N^*^** | | | | | |
| --- | --- | --- | --- | --- | --- | --- |
|  | **LAM**^#^ | **MTB/RIF** | **MTB/RIF + LAM**^#^ | **Smear**^#^ | **Smear + LAM**^#^ | **P-value^†^** |
| **Cape Town** | 99.0 (97.1-100.0) 100/101 | 95.6 (89.5-100.0) 43/45 | 93.3 (86.0-100.0) 42/45 | 98.2 (94.7-100.0) 55/56 | 98.2 (94.7-100.0) 55/56 | n/s |
| **Durban** | 97.9 (95.0-100.0) 93/95 | 95.7 (89.8-100.0) 44/46 | 97.7 (93.2-100.0) 42/43 | 98.1 (94.6-100.0) 53/54 | 94.2 (87.9-100.0) 49/52 | n/s |
| **Lusaka** | 87.1 (82.0-92.3) 142/163 | 90.2 (84.1-96.3) 83/92 | 80.4 (72.3-88.5) 74/92 | 90.1 (83.2-97.1) 64/71 | 83.1 (74.4-91.8) 59/71 | n/s |
| **Mbeya** | 89.7 (78.6-100.0) 26/29 | 87.5 (71.3-100.0) 14/16 | 68.8 (46.0-91.5) 11/16 | 69.2 (44.1-94.3) 9/13 | 69.2 (44.1-94.3) 9/13 | n/s |

^*^Denominator is the number of TB culture negative patients in the indicated group. Parent study [^2^](#_ENREF_2) involved randomisation to either Xpert MTB/RIF or smear-microscopy testing hence reduction in numbers.

^†^P-values shown are between diagnostic test (or test combinations) for each patient group (rows) and are marked with an asterisk (^*^ or ^*1^) to indicate tests compared.

^#^Specificity of LAM was higher in Cape Town and Durban compared to Lusaka and Mbeya (Cape Town/Durban vs. Lusaka p<0.001, Cape Town vs. Mbeya p=0.01, Durban vs. Mbeya p=0.04); specificity of LAM combined with Xpert MTB/RIF was higher in Cape Town and Durban compared to Lusaka and Mbeya (Cape Town vs. Lusaka p=0.05, Cape Town vs. Mbeya p=0.01, Durban vs. Lusaka p=0.007, Durban vs. Mbeya p=0.001); specificity of smear microscopy was higher in Cape Town and Durban compared to Mbeya (both comparisons p<0.001); specificity of LAM combined with smear was higher in Cape Town and Durban compared with Lusaka and Mbeya (Cape Town vs. Lusaka p=0.005, Cape Town vs. Mbeya p<0.001, Durban vs. Lusaka p=0.06, Durban vs. Mbeya p=0.009)

**Table S5C. Specificity of different diagnostic tests alone or in combination in HIV-infected patients culture-negative for tuberculosis and without clinical TB^¶^ stratified by study site.**

| **Site-specific performance (HIV-infected only)** | **Specificity (95% Confidence interval) of diagnostic test or test combination, n/N**^§^ | | | | | |
| --- | --- | --- | --- | --- | --- | --- |
|  | **LAM** | **MTB/RIF** | **MTB/RIF + LAM** | **Smear** | **Smear + LAM** | **P-value^†^** |
| **Cape Town** | 98.9 (96.8-100.0)^#^ 91/92 | 100.0 (91.4-100.00 41/41 | 97.6 (92.8-100.0) 40/41 | 98.0 (94.2-100.0) 50/51 | 98.0 (94.2-100.0) 50/51 | n/s |
| **Durban** | 98.5 (95.7-100.0) 67/68 | 100.0 (89.3-100.0) 32/32 | 100.0 (88.7-100.0) 30/30 | 100.0 (91.4-100.0) 39/39 | 97.4 (92.3-100.0) 37/38 | n/s |
| **Lusaka** | 92.3 (86.4-98.2) ^# *^ 72/78 | 100.0 (92.4-100.0)^* *1^ 47/47 | 89.4 (80.5-98.2)^*1^ 42/47 | 96.8 (90.6-100.0) 30/31 | 93.5 (84/9-100.0) 29/31 | ^*^p=0.05, ^*1^p=0.02, |
| **Mbeya** | 90.5 (77.9-100.0)^#^ 19/21 | 100.0 (77.2-100.0) 13/13 | 84.6 (65.0-100.0) 11/13 | 100.0 (67.6-100.0) 8/8 | 100.0 (67.6-100.0) 8/8 | n/s |

^¶^Reference standard “positive” includes sputum TB culture-positive patients together with those diagnosed with clinical TB (N=127). Clinical TB includes all culture-negative patients given anti-tuberculosis treatment based on either a sputum-positive Xpert MTB/RIF (n=14), smear microscopy (n=11), Chest x-ray suggestive of TB (n=96) or clinical suspicion alone (n=6).

^§^Denominator is the number of TB culture negative patients without clinical TB in the indicated group. Parent study (ref) involved randomisation to either Xpert MTB/RIF or smear-microscopy testing hence reduction in numbers.

^†^P-values shown are between diagnostic test (or test combinations) for each patient group (rows) and are marked with an asterisk (^*^ or ^*1^ or ^*2^ or ^*3^) to indicate tests compared.

^#^Specificity of LAM was higher in Cape Town and Durban compared with Lusaka and Mbeya and ^#^ indicated p<0.05

**Table S6.** Changes in 2-month and 6-month TB-related morbidity indices in patients treated for TB according to baseline culture status, stratified by LAM result.

|  | **TBscore** | | |
| --- | --- | --- | --- |
|  | **LAM strip positive** | **LAM strip negative** | **P-value** |
| **Two months (TBscore N=174)** | n=20 | n=154 |  |
| Median score (IQR) in patients initiated on treatment | 2 (0.5-3) | 2 (1-4) | 0.204 |
| Culture-positive (N=96, Lam positive [n=16], Lam negative [n=80]) | 1.5 (0.5-3) | 3 (1-4) | 0.204 |
| Culture-negative or contaminated (N=78, Lam positive [n=4], Lam negative [n=74]) | 2.5 (1-3) | 2 (2-3) | 0.693 |
| Median per patient change in score (IQR) since recruitment in patients initiated on treatment | 4.5 (2.5-5) | 3 (1-5) | 0.264 |
| Culture-positive (N=95, Lam positive [n=16], Lam negative [n=79]) | 5 (2.5-5) | 3 (1-5) | 0.169 |
| Culture-negative or contaminated (N=76, Lam positive [n=4], Lam negative [n=72]) | 4 (3-5.5) | 4 (2-5) | 0.690 |
| **Six months (N=160)** | n=13 | n=147 |  |
| Median score (IQR) | 3 (3-3) | 3 (1-4) | 0.575 |
| Culture-positive (N=86, Lam positive [n=9], Lam negative [n=77]) | 3 (3-3) | 3 (1-4) | 0.959 |
| Culture-negative or contaminated (N=74, Lam positive [n=4], Lam negative [n=70]) | 3.5 (2.5-4) | 3 (2-4) | 0.341 |
| Median per patient change in score (IQR) from month 0 (N=157) | 4 (3-5) | 3.5 (2-5) | 0.596 |
| Culture-positive (N=85, Lam positive [n=9], Lam negative [n=76]) | 4 (3-5) | 3 (1-5) | 0.136 |
| Culture-negative or contaminated (N=72, Lam positive [n=4], Lam negative [n=68]) | 3 (1.5-4.5) | 4 (2.5-5) | 0.468 |

Abbreviations: TB, tuberculosis; IQR, interquartile range;

**References**

1. Wejse C, Gustafson P, Nielsen J, et al. TBscore: Signs and symptoms from tuberculosis patients in a low-resource setting have predictive value and may be used to assess clinical course. *Scandinavian journal of infectious diseases* 2008; **40**(2): 111-20.

2. Theron G, Zijenah L, Chanda D, et al. Feasibility, accuracy, and clinical effect of point-of-care Xpert MTB/RIF testing for tuberculosis in primary-care settings in Africa: a multicentre, randomised, controlled trial. *Lancet* 2014; **383**(9915): 424-35.
